# Safety and Toxicity Issues of Therapeutically Used Nanoparticles from the Oral Route

**DOI:** 10.1155/2021/9322282

**Published:** 2021-10-28

**Authors:** Farzaneh Lotfipour, Shahriar Shahi, Afsaneh Farjami, Sara Salatin, Mohammad Mahmoudian, Solmaz Maleki Dizaj

**Affiliations:** ^1^Food and Drug Safety Research Center, Tabriz University of Medical Sciences, Tabriz, Iran; ^2^Department of Pharmaceutical and Food Control, Faculty of Pharmacy, Tabriz University of Medical Sciences, Tabriz, Iran; ^3^Dental and Periodontal Research Center, Tabriz University of Medical Sciences, Tabriz, Iran; ^4^Department of Endodontics, Faculty of Dentistry, Tabriz University of Medical Sciences, Tabriz, Iran; ^5^Pharmaceutical Analysis Research Center, Tabriz University of Medical Sciences, Tabriz, Iran; ^6^Faculty of Pharmacy, Tabriz University of Medical Sciences, Tabriz, Iran

## Abstract

The emerging science of nanotechnology sparked a research attention in its potential benefits in comparison to the conventional materials used. Oral products prepared via nanoparticles (NPs) have garnered great interest worldwide. They are used commonly to incorporate nutrients and provide antimicrobial activity. Formulation into NPs can offer opportunities for targeted drug delivery, improve drug stability in the harsh environment of the gastrointestinal (GI) tract, increase drug solubility and bioavailability, and provide sustained release in the GI tract. However, some issues like the management of toxicity and safe handling of NPs are still debated and should be well concerned before their application in oral preparations. This article will help the reader to understand safety issues of NPs in oral drug delivery and provides some recommendations to the use of NPs in the drug industry.

## 1. Introduction

**Figure 1:**
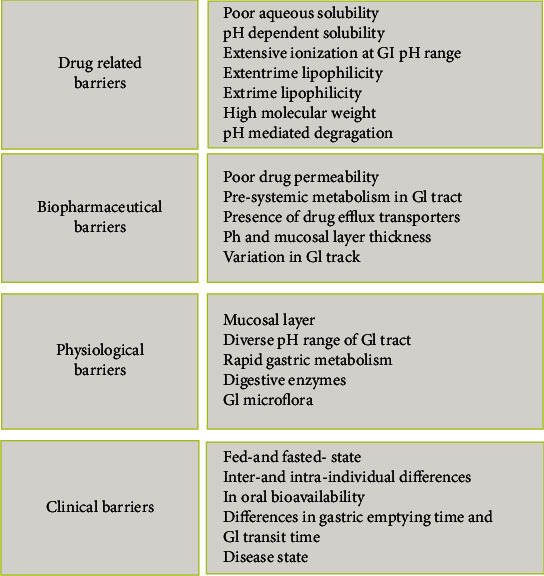
The most important challenges encountered in oral drug delivery.

The oral route is the most accepted and preferred route of administration as it provides patients many benefits such as no assistance, painless administration, cost-effectiveness, and flexibility in the design of dosage forms as compared to other routes like intravenous, intramuscular, and pulmonary [1, 2]. Regardless of the these benefits, the oral administration can have serious problems in delivering of therapeutic agents which are as follows: (a) low stability and solubility of drugs during the gastrointestinal (GI) tract, (b) inappropriate partition coefficient through the lipid membrane, (c) mucosal barrier and poor intestinal permeability, (d) first-pass metabolism, (e) undesired drug degradation in pH of the stomach or enzymatic degradation, and (f) P-glycoprotein- (P-gp-) mediated efflux. The presence of P-gp in the kidney, liver, and intestine results in the poor absorption of drug from the GI tract [3]. Many hydrophobic and hydrophilic therapeutic agents exhibit poor oral bioavailability owing to their low physicochemical (solubility, stability) and/or biopharmaceutical (permeability, metabolic stability) features [4, 5]. Moreover, biologics (e.g., peptides, proteins, and nucleic acids) are even more challenging for oral administration due to their large molecular weight, hydrophilicity (leading to low permeability), and poor chemical/enzymatic stability in the GI tract [6]. However, different species have physiological differences that should be considered in oral formulation design.  Figure 1 summarizes the most important challenges in oral delivery of therapeutics.

## 2. GI Tract Organization

**Figure 2:**
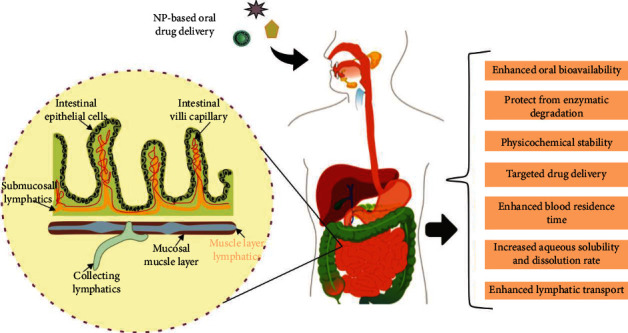
Oral administration of NPs: an emerging route to disease treatment.

Oral administration remains the preferred mode of delivery for drug formulations due to its convenience of dosing, cost-effectiveness, and high patient compliance [7]. The oral pathway takes advantage of the highly absorptive intestinal epithelium that provides an extensive surface area for drug absorption in the GI tract (300-400 m^2^) [8]. Each area of the GI tract provides protective, secretory, absorptive, and digestive functions [9]. Protein digestion begins in the stomach, by an enzyme called protease pepsin. The stomach pH varies with species as well as with diet and stage of ingestion. In humans, the gastric pH is between 1.2 and 2.0 in a fasted state and approximately 5.0 after food intake [10]. The small intestine is the site of digestion and absorption of the majority of nutrients including peptides, fats, and carbohydrates. Moreover, it exerts a wide variety of secretory and protective immune functions. The pH of the duodenum varies from 6 to 7 in humans [11]. As shown in Figure 2, the intestinal epithelium mainly consists of microvilli. Microvilli is composed of absorptive enterocytes, mucus secreting goblet cells, and specialized lymphoid structures, called Peyer's patches, covered with microfold cells (M cells) that have high uptake activity [12, 13].

The intestinal epithelial cells are joined with each other by tight junctions which constitute a physical barrier, selecting what can/cannot diffuse through the mucosal barrier due to reach the systemic circulation. The epithelial cells are covered by a thick mucosal layer which provides a dynamic barrier trough the intestinal tract. Mucus is continuously secreted by specialized mucus-secreting goblet cells and has been reported to be efficient in trapping larger particles. It contains mucopolysaccharides and glycoproteins, preventing luminal bacteria to reach the enterocyte surface. The mucosal layer is made of two layers which vary in thickness: a firmly adhered mucus gel layer attached to the mucosal epithelium and one layer of loosely attached mucus layer [14]. The total thickness of mucus (loosely adherent plus firmly adherent layers) ranges from 50 to 450 *μ*m in the human GI tract depending upon the location [15, 16]. While most absorption occurs in the upper small intestine, the distal small intestine and colon exhibit specialized capacities in absorbing water, B vitamin, and fatty acid [17].

## 3. Interaction of NPs with the GI Tract

In recent years, NPs have increasingly been explored to overcome GI tract barriers and improve the oral bioavailability by taking the excellent features of the GI tract and overcoming the problems like low oral solubility and bioavailability of drugs [18]. NPs are ultrafine colloidal particles that range from about 1 to 1000 nm in diameter and show different properties compared to their source materials. Therapeutic agents can be loaded intra NPs or incorporated via adsorption or chemical conjugation to the surface [19]. NP-based formulations offer a potential benefit for providing targeted and/or localized oral drug delivery. In this regard, NPs can be designed to target a particular region or specific cells within the GI tract [20]. Several oral nanosuspension-based products are now on the market that improves the dissolution or absorption characteristics of drugs [21]. Taking into consideration the physiological structure of the GI, it seems that the NP absorption takes place in the small intestine. Nevertheless, NPs have to overcome different GI barriers before they can be penetrated across the intestinal epithelium like mucosal layer and tight junctions in the intestinal epithelial cells. There is a much thinner firmly adherent mucus layer in the small intestine which offers the major site for the absorption of NPs [22]. Different strategies such as modified NPs possessing mucoadhesive [23], mucuspenetration [24], and/or mucolytic [25] characteristics have been proposed to overcome this barrier. Mucolytic NPs are able to directly disrupt the mucu*s* layer by mucolytic agents, thereby exposing the intestinal epithelium. This phenomenon promotes the NPs uptake and also facilities bacterial translocation, which will lead to infections. In the absence of mucin, the cell surface is exposed to the harsh environment of the GI tract, which leads to further damage.

**Figure 3:**
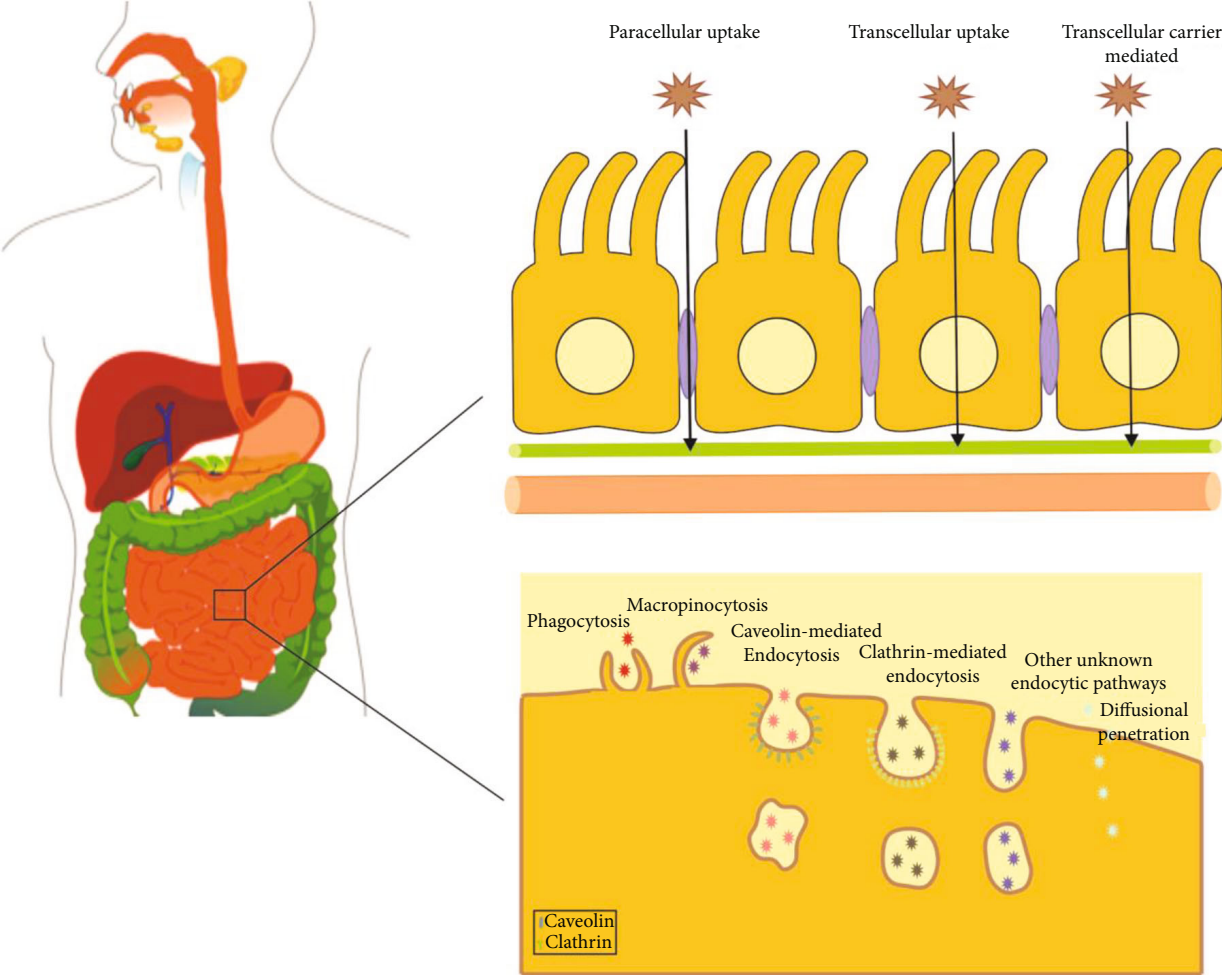
Cellular uptake mechanisms implemented by NPs for improving the oral bioavailability of drugs.

Potential outcomes of NPs ingestion include absorption, by which NPs can enter the systemic circulation and reach to other organs, interaction with the mucosal layer including physical effects on motility, and effects on luminal components, including the mucosal layer and the GI tract microbiome which play key roles in normal gut physiologic, immune, and metabolic activities [26]. As shown in Figure 3, NPs are able to pass through the epithelial barrier either by paracellular or transcellular pathway. In the transcellular pathway, NPs pass directly through the intestinal cells.

Cellular uptake can occur via different endocytic mechanisms where the NPs are internalized at the apical cell membrane, located within vesicle*s*, transported across the epithelial cell*s*, and released at the basolateral side of the cells [27]. The paracellular route exhibits the passage between the cells, via the tight junctions [28, 29]. Phagocytosis is a receptor-mediated mechanism involving ATP-dependent steps by which NPs are engulfed by the cellular membrane. However, this mechanism is specifically seen in the M cells due to a lack of a normal intestinal mucosal barrier and the presence of a scant glycocalyx which allows the transport of NPs. For this, the translocation of NPs across the M cells is higher than that of the enterocytes. The effective size cutoff of this process has been determined to be between 20 and 500 nm [30]. Micropinocytosis is a type of endocytosis in which the fluid-containing NPs are engulfed by the cellular membranes without any receptor-mediated mechanism. In other mechanisms, the cellular membrane forms pits in an ATP-dependent manner. When cellular uptake is a receptor-mediated process, it is referred to as clathrin mediated endocytosis, while when the mechanism is not receptor-mediated, it is referred to as caveole-mediated endocytosis. The paracellular pathway is not the main mechanism for the transport of NPs due to the small surface area of <1% of the total intestine and very limited intercellular space (3 to 10 A° of diameter) [31]. However, various types of cell-penetrating mediators and/or absorption enhancers can be used to facilitate the transport of NPs via the paracellular space [32, 33].

## 4. Toxicological Concerns with Oral NPs

With the improvement of NPs translocation, some new nanosystems are gradually being designed, which protects therapeutic agents from inactivation by acid and enzymatic barriers of the GI tract. Although the scientific understanding of NPs effect on the GI tract has not yet reached a reliable grade, novel NPs are suggested to clinical application. Despite the large number of NPs used in oral products, there is a relatively low knowledge on the possible toxic effects of NPs on the GI tract. The toxic effects of NPs via oral exposure may occur both locally via direct interaction with the intestinal epithelial cells or systemically, after they reach into the systemic circulation. Overall, the interactions between GI tract and NPs has been reported to lead the changes of mucus, epithelial cell integrity, tight junction, gut microbiota, and induce immune responses [34, 35]. In fact, both food and the processes that break down food ingredients (e.g., physical forces, digestive enzyme, osmotic concentration, and pH gradients) may alter the naive properties of NPs, thereby affecting their biological reactivity and toxicological effects [36]. In the preclinical development phase, the choice of appropriate experimental models is essential for the preparation of new oral NPs. Cell culture models are the most important in vitro test platform to predict interactions between the GI tract and drug delivery systems, providing valuable insights into the molecular mechanisms underlying the absorption and toxicity of NPs. Besides, cell culture-based approaches will need to choose the most appropriate formulations for further in vivo studies [36, 37]. Different culture methods have been suggested due to evaluate the effects of NPs on the cell viability of the GI tract. The most common cell culture models to evaluate the molecular mechanisms of toxicity are the cell-based Caco-2 systems [38]. In some cases, multicellular cultures have been suggested to better mimic in vivo GI situations, including conditions of inflamed mucosa. These models have reported to be more predictive of in vivo responses, susceptibility to the NPs injuries compared to the cellular monolayers, and can consist of microfold cells [39], mucus secreting goblet cells [40], and even immune-competent macrophages and dendritic cells [41]. Of note, coadministration of NPs with food matrix components or GI simulated biofluids can be used to provide more meaningful in vitro conditions and to examine the effect of protein corona on the absorption, metabolism, and toxicological behavior of NPs. Preliminary and complementary animal studies can also be applied for the assessment of the toxicity of orally ingested NPs [42]. In vivo studies can provide valuable data regarding toxicokinetics of NPs in the GI and extraintestinal tissues as well as toxicodynamic behaviors depending on their physicochemical characteristics [36]. On a physiological basis, the toxicity of NPs can be determined from pathological changes, including functional damage and morphologic changes. Histological study should be the initial step in which GI tract microvilli and epithelial atrophy [43] are evaluated using microscopic examination, and mast cell counts in gastric and/or duodenal tissue biopsies [44] are determined. More importantly, the toxicity of NPs can be influenced by their physicochemical characteristic such as composition, size,and charge, surface chemistry, or, in turn, by properties acquired through the GI tract transit. The physicochemical characteristics of NPs should be tailored to have lower nanotoxic effect on the cells. Other factors influencing the toxicological effects of NPs including GI transit time, nutritional status, meal quality, pH gradients, intestinal microflora, and amount of mucosal and enzymatic secretions may also affect the reactivity of NPs. The biological fate of degradation products of NPs is a major concern to our health. Many studies regarding the toxicity of NPs exhibited that the most commonly used materials to develop NPs are biodegradable and biocompatible. However, the cellular accumulation of a large number of NPs composed of biodegradable materials can cause severe cellular toxicity. It has been shown that extent of NP degradation depends on the GI tract conditions, e.g., pH or ionic strength. The toxicological effects also depend on the composition of the NPs core. The toxicity of NPs is largely related to the formation of free radicals (e.g., reactive oxygen and nitrogen species (RONs)). Excessive accumulation of RONs can damage to biomolecules like DNA, proteins, lipids, and carbohydrates. NPs may involve reactions that either stimulate RONs production or block the cellular RON-eliminating function. Such toxicity results from a combination of this process [45]. Metal-ion leakage from the core is the most common cause of the toxic effects of NPs. Some metal ions, like Ag and Cd, are toxic in fact, causing damage in living cells. Other metal ions, like Fe and Zn, are useful in biological systems, while, at high concentrations, they can lead to disturbance of cellular pathways and cause high toxicity. This process can be reduced by coating the core of NPs with thick polymer shells, or by using nontoxic chemicals for NPs synthesis. Core composition can be modified by adding other metals, resulting in increased chemical stability against NPs degradation and leakage of metal ions into the human body [46]. The size of NPs is one of the most important parameters affecting their oral toxicity [47]. The large surface area to volume ratio of NPs aids their harmful interactions with the biological systems, modifying cellular uptake [48]. According to the previous studies, smaller particles may pass through the enterocyte cell membranes, causing membrane fluidity. This results in altered signaling or increased cytotoxicity. The cellular uptake of ultrafine particles by the GI tract can also stimulate phygaocytosis at the mucosal layer [49]. This promotes antigen-antibody mediated reactions and inflammatory responses. In contrast to the smaller particles, the cellular uptake of larger particles is significantly mediated by M cells, which are already specialized for this function [50]. Lamprecht and coworkers demonstrated a size-dependent interaction of NPs with the mucosal layer [51]. The toxicity issues of NPs depend also to a large extent on the surface charge of NPs. NPs with positive surface charge are generally more toxic than those with negative surface charge [52, 53]. A prominent toxic effect of positively charged NPs is explained by their ability to easily interact with the mucus layer and to enter epithelial cells as compared to the neutral and negatively charged NPs. Improved cellular uptake can be mediated by enhanced electrostatic attraction between the negatively charged cell membrane glycoproteins and positively charged NPs [54]. On the other hand, the chemical modifications of materials may completely change the physicochemical characteristics and also the toxicity of NPs [55]. Surface modification means introducing chemical functional groups to the NPs surface via covalent modification and/or noncovalent modification by adsorption of biologically active molecules (e.g., surfactants, enzymes, antibodies proteins, and nucleic acids) [56]. For example, NP surface and their charges can be tailored by grafting polyethylene glycol (PEG). The surface coating offers NPs to bind selectively with different types of cells and biological molecules and decreases the toxicological effects of NPs. Besides, the surface coating significantly affects the pharmacokinetics, distribution, and accumulation of NPs in the body. Notably, the solvents and reagents applied through the development of NPs are increasingly required to be as less toxic as possible and should be identified in the final oral product to have a quality agreement with the FDA limits [57]. For instance, long-term use of surfactants and permeation enhancers can cause damage to the intestinal epithelium or structural organization in the tight junctions, thereby allowing the translocation of pathogens and various toxins across the GI tract [58]. Although the toxic problems are often take place as a result of the use of some materials in the structure of NPs, the absorption and pharmacokinetics of drugs or excipients can be affected to a greater extent when incorporated into the NPs [59]. For this, pertinent toxicology-related studies of NPs oral exposure were identified in this paper, focusing on polymer-, lipid-, carbon-, metal-, and protein-based NPs.

## 5. Different Nanoparticulate Systems

### 5.1. Polymer-Based NPs

Polymer-based NPs have broadly been used for delivery of different types of therapeutics because of their smaller size, biocompatibility, sustained drug-release profile, and ease of fabrication. Although polymeric NPs have many advantages, they still have some drawbacks. The main limitation is that some preparation methods use toxic organic solvents that could be hazardous to the biological systems [29, 57]. Polymer-based NPs may consist of natural, synthetic, or semisynthetic polymers [48]. NPs composed of chitosan, and poly-lactic-co-glycolic (PLGA) polymers are among the most studied systems for oral drug delivery. Chitosan is a natural polymer composed of N-acetyl-D-glucosamine and D-glucosamine produced by the alkaline deacetylation of chitin. The mucoadhesive property of chitosan makes it one of the most commonly used polymers for developing oral drug delivery systems [29, 60]. However, due to the lack of toxicity studies and different biodistribution patterns of chitosan, it still is not approved by the FDA as an excipient in the drug delivery field [61]. Of note, the factors including the derivative-type and molecular weight of chitosan could be considered as the crucial parameters affecting its biodistribution following oral administration. The study of Chae and coworkers [62] indicated that chitosan polymers are not absorbed by the GI tract, while the absorption of oligomers of chitosan [63] and chitosan derivatives such as trimethyl chitosan [64] and N-acetylglucosamine was reported by other studies [65]. Regarding the molecular weight of chitosan, recent studies revealed that chitosan oligomers with molecular weight of 3.8 kDa have higher plasma concentration and oral absorption compared to the higher molecular weight one (230 kDa) [62]. Turning to the toxicity aspect of chitosan, it is reported that the molecular weight together with the deacetylation degree influences the toxicity of chitosan [66]. The resulting data demonstrated that the toxic effects are more associated with the molecular weight and the concentrations of chitosan at high deacetylation degree of the polymer. The lethal dose of 50% (LD50) of chitosan was found 16 g/kg following its oral administration in mice, and a safety dose up 4.5 g/day was reported to chitosan after oral administration in humans. However, symptoms such as mild nausea and constipation were appeared after taken regularly for 12 weeks [57]. Considering the oral toxic aspect of chitosan NPs, different derivatives of the polymer were evaluated. According to the study conducted by Yin et al. [67], thiolated trimethyl chitosan NPs presented a lack of toxicity compared to the polymer solution. While the study of Zheng et al. [64] indicated slight toxic symptoms at high doses of thiolated trimethyl chitosan NPs, Sonaje and coworkers [64] investigated the toxicity of NPs made of poly-g-glutamic acid and chitosan in the presence of MgSO4 and sodium tripolyphosphate. The NPs were pathologically well tolerated after taken for 14 days, and no relevant pathological changes and inflammatory reactions were also observed. Meanwhile, Du et al. [68] evaluated the toxicity of decanoic acid-grafted oligochitosan NPs in rats. Histopathology studies represented that the villus structure of the intestinal epithelium was normal, and there were no significant inflammatory reactions. Another search group performed the toxicity study of NPs of alginatedextran sulfate core complexed with a bilayer of chitosan-PEG and albumin coat. The resulting data showed that the NPs were well tolerated with no signs of inflammatory reactions after twice daily administration of these NPs over 15 days in rats [69].

In addition to the natural polymers like chitosan, synthetic polymers also extensively have been used for the development of new delivery systems for oral administration of drugs. PLGA is a synthetic polymer that mainly due to its biodegradability and biocompatibility, and sustained drug-release profiles are commonly used for the oral drug delivery applications. In vivo/in vitro studies revealed no toxicity concerns for PLGA NPs and chitosan-modified PLGA NPs [70, 71]. It was reported that PLGA NPs made using dodecyl dimethylammonium bromide (a quaternary ammonium salt) was safe, and orally administered NPs were effective at a 50% lower dose compared with the intravenous route [72]. In another study, Jain et al. [73] reported that PLGA NPs as a drug carrier significantly reduce the hepatoxicity of tamoxifen compared with the free drug solution in rats.

Dendrimers are another kind of polymeric NPs that only to some extent has been used for oral drug applications. These drug carriers composed of a hydrophobic core and a hydrophilic periphery, constituted by polymeric branches [74]. Notably, the physicochemical properties of the dendrimers including surface charge, length, and termination play important roles in the toxicity of the particles. It was reported that the anionic NPs are well tolerated and less toxic compared with the cationic ones. However, the surface modification of cationic residues with noncharged groups improved their safety and uptake by the epithelial cells. Meanwhile, smaller dendrimers were safer than the larger ones [75].

### 5.2. Lipid-Based NPs

Different types of lipid-based NPs including liposomes, transfersomes, solid lipid NPs (SLNs), nanostructured lipid carriers (NLCs), and self-emulsifying drug delivery systems have been used to improve the oral bioavailability of different therapeutics. Among them, liposomes, SLNs, and NLCs are commonly used lipid-based NPs, while the use of other NPs as oral drug carriers has been very limited [76, 77]. Liposomes consist of an inner aqueous core surrounded by a lipid bilayer while lipid NPs consist of a solid lipid core surrounded by a lipid monolayer [78]. Because of their unique structure, liposomes have the possibility of incorporating both hydrophilic and hydrophobic drugs. As liposomes are mainly composed of natural phospholipids, they are typically considered pharmacologically inactive with minimal toxicity. However, there are some disadvantages associated with liposomes including low solubility, high cost, and a short half-life [29]. SLNs are biocompatible colloidal particles composed of a hydrophobic core of lipid which remains solid at body temperature. SLNs are able to incorporate hydrophobic drugs with high encapsulation efficiency and show excellent physical stability. The main limitations of SLNs are high tendency towards aggregation and the risk of drug expulsion due to the crystallization process during storage [77]. NLCs are prepared by mixing solid lipids with spatially incompatible liquid lipids, resulting in structures with excellent advantages including improved drug loading, biocompatibility, biodegradability, and controlled drug release. Of note, NLCs exhibit lower drug leakage during storage period than SLNs [59]. Cytotoxicity study of SLNs showed 90% cell viability in Caco-2 cells [79], as well as tripterine-encapsulated NLCs showed greatly decreased cytotoxicity compared with the free drug solution [80]. In vivo toxicology of SLNs represented no evidence of damage of the intestinal epithelium following oral administration. Similarly, less GI toxicity was reported for tripterygium-loaded SLNs compared with the drug solution [81]. The study performed by Lv et al. [82] showed that the self-double-emulsifying drug delivery systems cause no toxicity in Caco-2 cells. However, the histopathologic studies demonstrated that these systems could cause mucosal damage in the rat intestine. Of note, recent studies have explored that the use of high cationic lipids is associated with an increase in in vitro and in vivo cell toxicity such as cell shrinking, vacuolization of the cytoplasm, and detrimental effects on key cellular proteins [83]. Here, toxicity issue is mainly due to the interaction of positively charged head group in cationic NPs with negatively charged components in the cells [84].

### 5.3. Carbon-Based NPs

A variety of carbon-based NPs including carbon nanotubes (CNTs), carbon nanohorns, and graphene oxide have been developed for oral administration. CNTs are cylindrical structures that belong to the fullerene family and may be constructed as single-walled CNTs or multiple-walled CNTs [85]. CNTs take advantages such as high surface area, conductivity, tensile strength, and potential absorption capabilities [86]. Besides, CNTs can provide the possibility of controlled and site-specific drug delivery. The biocompatibility and biodegradability of these systems can be enhanced by surface functionalization [87]. The main disadvantages of CNTs are poor water solubility in aqueous medium and very high cost of production. The results of toxicological studies have reported a contradictory overview of the utility of CNTs via the oral route. Some of these works demonstrated acute toxicity and genotoxicity concerns, while other studies exhibited the safety of CNTs for oral drug delivery applications. The metallic impurity contaminants strapped inside the CNTs may be the main cause of toxicity. Local intestinal damage was observed after oral exposure to CNTs. In a recent work, multiple necrotic foci were observed in the small intestine after a 30-day exposure to CNTs in mice. It may be for this reason that mechanical damage to the enterocytes is mediated by CNTs. The authors found that a 6-month chronic exposure to CNTs results in a dose-dependent decrease in the number of small intestinal villi [88]. Another type of carbon-based nanomaterials is carbon nanohorns with a size range of 80-100 nm. Carbon nanohorns are cone-shaped hornlike structures, which are made of graphene sheets in the form of single-walled carbon nanomaterials [89]. The study performed by Miyawaki et al. [90] demonstrated the safety of carbon nanohorns at a dose of 2000 mg/kg for peroral administration, as the bodyweight of the rats remained normal over the 2-week test period. Carbon nanohorns had high purity in comparison with the CNTs due to no need for metal catalysts during the preparation process. Graphene oxides are another type of carbon-based NPs that have attracted much attention for oral drug delivery. These drug carriers are constructed as single atom thick carbon sheets with pH-dependent properties that are produced by the harsh oxidation of crystalline graphite. In a recent study, Rahmanian et al. [91] investigated graphene oxides as efficient platforms for oral delivery of quercetin. Yang and coworkers [92] investigated the oral toxicity of graphene oxides and PEG-tailored graphene oxides at a dose of 100 mg/kg to mice. Analyzing after 30 days revealed that the PEG-tailored graphene oxides were not toxic at the tested dose. However, more studies must be conducted to better understanding the toxicity of graphene oxides for oral drug delivery.

### 5.4. Silicon-Based NPs

Silicon-based NPs are biocompatible and biodegradable drug delivery systems with the chemically inert entities. In addition to the ease of fabrication process, the physicochemical properties of these particles are tailorable that make them an optimal alternative for drug delivery via different routes. Difficulty in controlling particle size and morphology are some of important disadvantages of silicon-based NPs [54]. Silicon-based NPs are generally prepared as mesoporous or nonporous NPs. This provides the possibility of drug loading into the pores of the mesoporous NPs or physically adsorbed on the surface of nonporous NPs. In recent years, silicon-based NPs have attracted great attention as new materials for developing oral formulations. However, more studies should be done to cover all toxicological concerns of these NPs for oral drug delivery.

#### 5.4.1. Nonporous Silica NPs

Nonporous silica is majorly used as food and pharmaceutical additive, while its capacity to use as drug nanocarrier has been rarely investigated [93]. In this regard, subchronic toxicity of oral dosage of two silica NPs including nanostructured silica (NM-202) with size of 15-25 nm and synthetic amorphous silica with size of 7 nm was daily measured after oral administration. Daily dose of NM-202 and amorphous silica was 100 to 2500 milligram per each kilogram of body weight for 28 days and continued up to 84 days for the highest doses. Althoug, no signs of significant increase in the silica level in different tissues were detected after 24 days, the silica was accumulated in spleen after 84 days of amorphous silica administration. Furthermore, in liver samples, a remarkable increase in the level of fibrosis-related genes along with the liver fibrosis was observed as a consequence of NM-202 administration after 84 days [94]. Although this study showed that silica NPs are not toxic even at high doses, a need to more studies to find the reason of liver fibrosis and silica accumulation in spleen is still remained. In consistent with the previous study, the toxicity of oral dosage of silica NPs was detected in various body tissues such as kidney, lung, liver, and testis in the recent study performed by Hassankhani et al. [95]. In this research, mouses were orally gavaged with silica NPs (10-15 nm) at daily dose of 333 mg/Kg for 5 days. At the end of the experiment, one mouse died, and the symptoms of appetite loss, vomiting, and severe loss of energy were observed in others. Furthermore, other signs of toxicity of silica NPs such as alteration in triglyceride, cholesterol, albumin, total protein, and high and low-density lipoprotein were observed only after 5 days of administration.

#### 5.4.2. Mesoporous Silica NPs

The remarkable potential of mesoporous silica NPs as a multifunctional drug delivery system has been proven since its first development in 2001 [96]. Fu et al. [97] studied the pharmacodynamics and toxic effects of mesoporous silica NPs in mice via oral administration of 50, 500, and 5000 mg/kg of body weight. No death of any mice and also no symptoms of appetite and weight loss were observed after 24 h administration. Moreover, it was revealed that the absorption of mesoporous silica NPs was done through the portal vein in the liver. More than 80% of the primary dose of mesoporous silica NPs was excreted in feces. So, according to the acceptable tissue biocompatibility of mesoporous silica NPs, the safe route of NPs delivery was suggested to be the oral route.

#### 5.4.3. Mesoporous Silicon NPs

Mesoporous silicon is one of the most potent NPs to use as nanocarriers for both small molecules and macromolecules. The surface modification of mesoporous silicon can be easily performed to create hydrophobic or hydrophilic properties. Also, chemical conjugation with chitosan as a biopolymer can be used to modify the mesoporous silicon surface [98]. Several studies have investigated the cytotoxicity of mesoporous silicon on intestinal Caco-2 cell line and mucus-producing goblet cells HT29 [99, 100]. In these projects, the various chemistry of the mesoporous silicon surface such as thermally hydrocarbonized, thermally oxidized, and undecylenic acid-modified particles in the range around 150-200 nm has been evaluated. The resulting data demonstrated that the viability of both HT29 and Caco-2 cells was preserved up to 80% even after 24 h exposure to thermally oxidized mesoporous silicon; therefore, thermally oxidized mesoporous silicon induced the least cytotoxicity. However, slight toxic effects were observed by thermally hydrocarbonized mesoporous silicon at concentrations more than 100 *μ*g/ml [99]. The viability of both HT29 and Caco-2 cells was maintained more than 80% as a result of exposure to undecylenic acidmodified mesoporous silicon NPs at all concentrations and at both time periods, except after 12 h incubation of all concentrations of undecylenic acidmodified mesoporous silicon with Caco-2 cells. However, the viability of both cell lines significantly increased after exposure to undecylenic acid mesoporous silicon NPs modified by chitosan for both incubation time. According to the information obtained by these studies, the toxic effect of mesoporous silicon NPs on cells depends on the surface features of the mesoporous silicon NPs. In another study, it was found that thermally hydrocarbonized mesoporous silicon NPs at concentrations up to 250 *μ*g/ml had no significant cytotoxicity on Caco-2 cells, and also the size of NPs in the range around 97 to 188 nm had no significant role in cytotoxicity on Caco-2 cells. Moreover, the biodistribution of 18F-labeled thermally hydrocarbonized mesoporous silicon NPs was studied in vivo after its oral administration. The detection of trace amount of radioactivity in systemic circulation showed that the mesoporous silicon NPs remained in the GI tract [101].

### 5.5. Metallic NPs

Gold NPs (AuNPs), silver NPs (AgNPs), and supramagnetic metal oxides (iron oxides: Fe2O3 or Fe3O4), are the most popular candidates of metal NPs. In addition to the use of metallic NPs as drug delivery systems, they are majorly utilized for imaging.

#### 5.5.1. AuNPs

Colloidal AuNPs are extensively applied in medical applications as imaging and therapeutic agents, particularly as drug delivery systems. AuNPs are easily prepared with intended features including small size, proper shape, low toxicity, and suitable surface functionalities. Among the metallic NPs, AuNPs are best candidate to use as drug delivery systems. Limited studies have been devoted to evaluate oral delivery systems and toxicity effects of AuNPs. Clinical improvement without toxicity was investigated with a colloidal gold-based tablet after oral administration [102]. The accumulation site of AuNPs with different size (4, 10, 28, and 58 nm) was investigated by Hillyer and coworkers [103]. They found that AuNPs with size of 4 nm had potential to penetrate across the GI tract easily and to accumulate highly in the brain, spleen, liver, kidney, and lungs in comparison with other particle sizes. In another study, AuNPs with a smaller particle diameter revealed an increased rate of absorption by intestinal epithelium cells and decreased cellular accumulation. However, AuNPs demonstrated the potential cytotoxicity in the intestinal epithelial cells by depolarization of mitochondria membranes. This study can offer important insights into the relationship between the particle diameter of AuNPs and their GI tract absorption and subsequently cytotoxicity [104]. The toxic effects of chitosan-reduced AuNPs in rats after oral administration were studied during 28 days by Pokharkar et al. [105]. No signs of subacute toxicity such as alteration in organ and body weight, food administration, hematological factors, histopathological, and clinical sign changes were found. Furthermore, the dose of more than 2000 mg/kg of body weight was proved to be the median lethal dose of chitosan-reduced AuNPs in rats. In another study, the toxicity of AuNPs with the size of 13.5 nm at two oral doses including 137.5 and 2200 *μ*g/kg was assessed. According to the results, a notable decrease in the body weight with no significant toxicity was observed after oral administration of low dose AuNPs. However, oral delivery of high dose AuNPs caused a significant decrease in count of red blood cells and increase in the accumulation of AuNPs in spleen after 28 days. Therefore, in the development of NP based oral delivery systems, not only the size and surface coating but also the dose of AuNPs are important parameters [106].

#### 5.5.2. AgNPs

AgNPs refer to the metallic Ag with a size scale between 1 and 100 nm which are commonly obtained from inorganic salts. AgNPs can be synthesized by conventional techniques as well as an alternative technique so-called green synthesis [107]. Depending on the preparation method, AgNPs are varied in architecture and structure from oval, triangular, hexagonal shape to nanowire forms [108]. Although AgNPs exhibit a promising future in the field of drug delivery, they can create reactive oxygen species and free radicals which cause apoptosis, resulting in cell death. The main entry route for AgNPs into the GI tract system is intentional ingestion. Upon oral administration, AgNPs can translocate through several compartments of the GI tract (e.g., mouth, stomach, and intestine). However, the fate of AgNPs after entering the GI tract is not yet known. It is noteworthy to say that the toxicity of AgNPs is significantly affected by the volume of distribution in the body. Variability in biochemical composition and pH within the GI compartments can affect the physicochemical characteristics of AgNPs (e.g., agglomeration and dissolution) as well as their bioavailability and toxicological features [109]. In biological environments, AgNPs may undergo a series of biochemical transformations to form secondary particles, and this may cause potential toxic effects on human health [110]. For example, toxic effects on a coculture of CaCo 2 cells and mucus producing cells were less pronounced with 200 nm AgNPs in comparison to 20 nm AgNPs [111]. On the other hand, oral exposure to AgNPs may affect the secretion of mucus, in both quantitative and qualitative terms. Mucus secretion in the ileum and rectum increased following subchronic (28 days) oral exposure to 60 nm AgNPs in rats. Moreover, changes in the amounts of neutral and acidic mucins and proportions of sulfated and sialylated mucins were observed [112]. Jeong's group [112] investigated that AgNPs are able to accumulate in the lamina propria in both the small and large intestine, in the tip of the upper villi, the ileum, and the protruding surface of the colon in a dose-dependent manner. In another research work, the resulting data demonstrated that AgNPs can be accumulated in the liver, blood, brain, and muscles [113]. AgNPs were shown to have toxic effects only at doses of 125 mg/kg and above. The lowest toxic effects of AgNPs at 125 mg/kg corresponded to increased cholesterol and cholestatic enzymes and were accompanied by biliary hyperplasia during a 90-day study [114]. This group also demonstrated cholestatic enzyme effects and slight hemoconcentration in rats given at dose of 300 mg/kg AgNPs for 28 days [115]. AgNPs were administered to mice at doses ranging from 0.25 to 1 mg/kg for 28 days. Adverse effects included a dose-dependent increase in both serum proinflammatory and anti-inflammatory cytokines. Moreover, there was a mild increase in B-cells and IgE [116]. Notably, only one rodent study investigated adverse effects of ingested AgNPs on the gut microbiota. Hadrup and coworkers [117] reported that there was no alterations in the balance and number of the two major bacterial phyla in the gut (Bacteroidetes and Firmicutes) when dosed with 14 nm Ag-polyvinyl pyrrolidone or silver acetate for 28 days. In pigs administered up to 40 ppm AgNPs (60-100 nm) in feed for 2 weeks, a decrease in intestinal coliforms was observed [118].

#### 5.5.3. Supramagnetic Metal Oxides

Due to the remarkable characteristics of supramagnetic metal oxide NPs such as uniform morphology, controllable size, surface enhanced Raman scattering, and strong plasma absorption, they are widely used in magnetic resonance imaging as contrast agents and even in drug delivery to develop magnetic responsive systems [119]. An important concern regarding the use of metal oxide NPs for in vivo applications is their toxicity to cause harmful effects on the living systems. In this regard, the in vivo study using female Wistar rat model was performed by Kumari et al. [120] to measure acute toxic effects of iron oxide (30 nm; Fe2O3-30) and iron oxide bulk (Fe2O3) after oral delivery. Besides, size and dose of NPs were investigated in this study. No significant alteration in the biochemical markers was observed after oral administration of bulk Fe2O3 while, Fe2O3-30 administration inhibited acetylcholinesterase enzyme in the red blood cells and brain as well as activated hepatotoxic marker enzymes in the serum and liver. These observations revealed that oral delivery of Fe2O3 NPs can lead to the inverse consequences in the biochemical profile. In another *in vivo* study, genotoxicity of Fe2O3-30 and Fe2O3 was investigated after oral administration of both NPs at doses of 500 to 2000 mg/kg to female Wistar rats [121]. It was detected that the biodistribution of Fe2O3-30 in various tissues and organs is highly depended on the size and dose of NPs. However, no significant genotoxicity of these particles was observed. It should be noted that, in the most studies, the biodistribution of NPs was considered, and limited studies were devoted to evaluate the toxicity and interaction of these particles with tissues. Furthermore, a lack of comprehensive studies evaluating chronic exposure effects or measurement of lethal dose is extensively felt. So, there is an extreme need to comprehensive studies to obtain better grasp of safety profile of NPs in biological barriers.

### 5.6. Protein NPs

Overall, self-assembled protein or mixture of proteins are able to form protein-based systems which can have various structures such as NPs, minifilms, minirods, films, hydrogels, microspheres, and cages [122, 123]. Protein NPs can be formulated using natural molecules like gelatin, albumin, whey protein, legumin, elastin, gliadin, zein, soy protein, and milk protein. Protein NPs can be incorporated into the biodegradable polymer-based microspheres for controlled and sustained drug release. Protein NPs have promising features like controllable size, ease of modification, biodegradability, biocompatibility, nonantigenicity, and great stability during storage conditions [124, 125]. The luminal fluid of the small intestine contains various enzymes, like pepsin, trypsin, nuclease, and carboxypeptidases which can typically degrade protein-based NPs and might even degrade the polypeptide surface coatings of NPs [126]. As an endogenous material, the use of albumin NPs has recently increased significantly for oral drug delivery. For example, no toxic effect of albumin NPs was detected on Caco-2 cells. Moreover, albumin NPs exhibited good permeation through the intestinal epithelial barrier [127]. In another research study, apotransferrin- and lactoferrin-based NPs have been used for oral delivery of doxorubicin. According to the resulting data, the use of transferrin NPs to doxorubicin oral delivery significantly decreased its heart and liver toxicity [128]. Although biocompatibility of the polypeptides and proteins used in nanosystems makes these delivery systems safe, extensive in vivo and in vitro studies are needed to assess their toxicity for several chronic therapies.

## 6. Conclusion

In recent years, nanotechnology has received much attention in the field of diagnostics and drug delivery. However, the number of nanotechnology-based systems that are actually marketed is minimal. This can be related to the absence of toxicity information on the potential hazards associated with these systems. Oral pathway has been reported as usually the safest administration route. However, the knowledge of the interaction and fate of NPs in the biological systems is negligible. The capability of NPs to cross the intestinal epithelial barrier and to enter the systemic circulation may pose a significant risk to public health. Different nanoparticulate systems possess unique physicochemical characteristics and therefore, special consideration needs to be given to each system. Therefore, the safety/toxicity assessment of NPs is required in order to have safe systems for efficient oral drug delivery.

## Data Availability

The data is available in pharmacy.
